# Association of body mass index and long-term mortality in patients from nationwide LIPIDOGRAM 2004–2015 cohort studies: no obesity paradox?

**DOI:** 10.1186/s12933-023-02059-0

**Published:** 2023-11-28

**Authors:** Tadeusz Osadnik, Dariusz Nowak, Kamila Osadnik, Marek Gierlotka, Adam Windak, Tomasz Tomasik, Mirosław Mastej, Beata Łabuz-Roszak, Kacper Jóźwiak, Gregory Y. H. Lip, Dimitri P. Mikhailidis, Peter P. Toth, Naveed Sattar, Marcin Goławski, Jacek Jóźwiak, Maciej Banach, the LIPIDOGRAM Investigators

**Affiliations:** 1 Department of Pharmacology, Faculty of Medical Sciences in Zabrze, Medical University of Silesia, Katowice, Poland; 2 Department of Pharmacology, Faculty of Medical Sciences in Zabrze, Medical University of Silesia, Jordana 38 St., 41-808 Zabrze, Poland; 3 Cardiology and Lipid Disorders Clinic, Independent Public Health Care Institution “REPTY” Upper Silesian Rehabilitation Centre, ul. Śniadeckiego 1, 42-600 Tarnowskie Góry, Poland; 4 Municipal Hospital, ul. Mirowska 15, 42-202 Czestochowa, Poland; 5 Department of Cardiology, Institute of Medical Sciences, University of Opole, Al. W. Witosa 26, 45-401 Opole, Poland; 6 Department of Family Medicine, Jagiellonian University Medical College, Bochenska 4 Street, 31-061 Kraków, Poland; 7 Mastej Medical Center, Staszica 17A St., 38-200 Jasło, Poland; 8 Department of Neurology, Institute of Medical Sciences, University of Opole, Oleska 48 St., 45-052 Opole, Poland; 9 Faculty of Health Sciences, Jagiellonian University Collegium Medicum, ul/Street: Piotra Michałowskiego 12, 31-126 Kraków, Poland; 10 Liverpool Centre for Cardiovascular Science, University of Liverpool, Liverpool, UK; 11 Liverpool Centre for Cardiovascular Science, Liverpool John Moores University, Liverpool, UK; 12 Liverpool Centre for Cardiovascular Science, Liverpool Heart and Chest Hospital, Liverpool, UK; 13 Danish Center for Clinical Health Services Research, Department of Clinical Medicine, Aalborg University, 9220 Åalborg, Denmark; 14 Department of Clinical Biochemistry, Royal Free Hospital Campus, University College London Medical School, University College London (UCL), Pond St., London, NW3 2QG UK; 15 Ciccarone Center for the Prevention of Cardiovascular Disease, Johns Hopkins University School of Medicine, Baltimore, MD 21287 USA; 16 Department of Preventive Cardiology, CGH Medical Center, 101 East Miller Road, Sterling, IL 61081 USA; 17 Institute of Cardiovascular and Medical Science, University of Glasgow, University Place, Glasgow, G12 8TA UK; 18 Department of Family Medicine and Public Health, University of Opole, Oleska 48 St., 45-052 Opole, Poland; 19 Department of Cardiology and Adult Congenital Heart Diseases, Polish Mother’s Memorial Hospital Research Institute (PMMHRI), Rzgowska 281/289, 93-338 Lodz, Poland; 20 Cardiovascular Research Centre, University of Zielona Gora, ul. Zyty 28, 65-046 Zielona Gora, Poland; 21 Department of Preventive Cardiology and Lipidology, Medical University of Lodz (MUL), Rzgowska 281/289, 93-338 Lodz, Poland

**Keywords:** Obesity paradox, Weight loss, Body mass index, Mortality, Primary care

## Abstract

**Background:**

An obesity paradox has been described in relation to adverse clinical outcomes (e.g., mortality) with lower body mass index (BMI).

**Aims:**

We sought to evaluate the association between BMI and weight loss with long-term all-cause mortality in adult populations under the care of family physicians.

**Methods:**

LIPIDOGRAM studies were conducted in primary care in Poland in 2004, 2006, and 2015 and enrolled a total of 45,615 patients. The LIPIDOGRAM Plus study included 1627 patients recruited in the LIPIDOGRAM 2004 and repeated measurements in 2006 edition. Patients were classified by BMI categories as underweight, normal weight, overweight and class I, II, or III (obesity). Follow-up data up to December 2021 were obtained from the Central Statistical Office. Differences in all-cause mortality were analyzed using Kaplan‒Meier and Cox regression analyses.

**Results:**

Of 45,615 patients, 10,987 (24.1%) were normal weight, 320 (0.7%) were underweight, 19,134 (41.9%) were overweight, and 15,174 (33.2%) lived with obesity. Follow-up was available for 44,620 patients (97.8%, median duration 15.3 years, 61.7% females). In the crude analysis, long-term all-cause mortality was lowest for the normal-weight group (14%) compared with other categories. After adjusting for comorbidities, the highest risk of death was observed for the class III obesity and underweight categories (hazard ratio, HR 1.79, 95% CI [1.55–2.05] and HR 1.57, 95% CI [1.22–2.04]), respectively. The LIPIDOGRAM Plus analysis revealed that a decrease in body weight (by 5 and 10%) over 2 years was associated with a significantly increased risk of death during long-term follow-up—HR 1.45 (95% CI 1.05–2.02, *p* = 0.03) and HR 1.67 (95% CI 1.02–2.74, *p* < 0.001). Patients who experienced weight loss were older and more burdened with comorbidities.

**Conclusions:**

Being underweight, overweight or obese is associated with a higher mortality risk in a population of patients in primary care. Patients who lost weight were older and more burdened with cardiometabolic diseases, which may suggest unintentional weight loss, and were at higher risk of death in the long-term follow-up. In nonsmoking patients without comorbidities, the lowest mortality was observed in those with a BMI < 25 kg/m^2^, and no U-curve relationship was observed.

**Supplementary Information:**

The online version contains supplementary material available at 10.1186/s12933-023-02059-0.

## Introduction

Overweight and obesity are recognized risk factors for diabetes mellitus (DM), myocardial infarction (MI), hypertension (HTN) and heart failure (HF), as well as cancers, which are the main causes of mortality in developed countries [1–6]. Despite the continuous increase in body mass index (BMI) in various populations over the last decade [7] and the well-documented negative effects of obesity on the risk of developing the abovementioned diseases, some doubts persist as to the relationship between BMI and patient prognosis [7, 8].

Lower mortality in overweight patients or patients living with obesity compared with patients with a BMI within the normal values was reported in subpopulations with atherosclerotic cardiovascular diseases (ASCVD), especially stroke, acute coronary syndromes (ACS), HF as well as DM [9, 10]. Thus, there is a paradoxical contradiction between the recommendations [11] to reduce body weight in the case of overweight and obesity and reports on the alleged protective effect of increased BMI in some groups of patients. Therefore, we aimed to assess the association between BMI and weight loss long-term all-cause mortality in a population of adult patients under the care of family physicians in Poland.

## Methods

### Design

We conducted a nationwide cohort study in which patients in 2004, 2006 and 2015 were recruited by a random sample of primary care practices and all-cause mortality was observed until the end of 2021.

### Study population

LIPIDOGRAM is a nationwide survey of cardiovascular risk factors carried out through primary care outpatient centers in Poland in 2004, 2006 and 2015–2016. The methodology of the LIPIDOGRAM2004, LIPIDOGRAM2006 and LIPIDOGRAM 2015 studies were described in detail elsewhere [12–14]. Briefly, physicians were selected randomly, using Medical Data Management Software. The number of physicians in each administrative region in Poland was selected in a manner proportional to the number of inhabitants. All consecutive patients aged ≥ 18 years were eligible for recruitment. In 2004, a total of 675 primary care physicians (PCPs) enrolled 17,522 patients in 444 cities. In 2006, 556 PCPs from 402 Polish cities recruited a total of 15,465 patients, while in 2015–2016 a group of 438 PCPs from 398 cities recruited additional 13,724 patients. After exclusion of doubled and incomplete records, 45,615 unique records were available for analysis. In conjunction with the LIPIDOGRAM 2004 and 2006 studies, the LIPIDOGRAM PLUS study encompassed 1627 patients who had repeated anthropometric and laboratory measurements in 2004 and 2006. Follow-up data were obtained from the Central Statistical Office using a unique identification number for each patient. The data were collected up to December 2021.

### Anthropometric measurements and physical examination

Height and weight measurements were carried out by nurses or physicians on patients in their underwear and barefoot. BMI was calculated by dividing body weight in kilograms (kg) by squared height in meters (m) (kg/m^2^). Waist circumference (WC) was measured at the midpoint between the lower margin of the ribs and the anterior superior iliac crest spine in centimeters (cm).

### Biochemical analyses

Blood samples were collected after fasting (> 12 h following last meal, there were no restrictions with regard to water intake). After centrifugation, blood samples were transferred to a core facility for processing. Biochemical analyses were performed within 12 h after blood sample collection in the core lab in Katowice, Poland. Serum concentrations of total cholesterol (TC) were measured using a photometric method. High density lipoprotein (HDL) cholesterol (HDL-C) and triglycerides (TG) were measured by an immunoseparation-based homogenous assay and colorimetric enzymatic test with glycerol-3-phosphate oxidase, respectively (DiaSys—Diagnostic Systems, Holzheim, Germany). Low density lipoprotein cholesterol (LDL-C) was calculated using the Friedewald formula (LIPIDOGRAM2004 and LIPIDOGRAM2006 surveys) or was measured directly (LIPIDOGRAM2015).

### Definitions

Patients within BMI categories of < 18.5, 18.5–24.9, 25–29.9, 30–34.9,35–39.99, and ≥ 40 kg/m^2^ were considered underweight, normal weight, overweight and living with obesity class I, class II and class III, respectively. Metabolic syndrome (MetS) was diagnosed according to the Joint Interim Statement Criteria (JIS) criteria [15]. Dyslipidemia was diagnosed by fulfilling at least one of the following criteria: increased LDL-C (> 115 mg/dl; > 3 mmol/l) or statin treatment. DM and HTN were diagnosed by the primary care physicians according to the contemporary guidelines [16–19].

### Statistical analyses

Continuous variables are presented as means and standard deviations (SD). The comparison of continuous variables across BMI categories was performed using the Kruskal‒Wallis test. The comparison of dichotomous variables was performed using the chi-square test. Non-adjusted and adjusted associations between mortality and BMI categories (underweight, normal weight, overweight, class 1 obese, class 2 obese, class 3 obese) were assessed using a Cox regression model. In the latter, in addition to BMI categories, age, sex (male, female), education (higher/secondary vs primary/vocational), smoking, place of residence (urban *vs* rural), DM, HTN, and dyslipidemia were included. As BMI categories are to some extent arbitrary, we visualized associations between the hazard ratio of mortality and BMI by plotting adjusted and unadjusted Cox regression models using a penalized spline basis.

We also used a Cox regression model with penalized splines to assess the association between change in body weight and mortality in patients from the LIPDIOGRAM PLUS sub-study for whom we had repeated anthropometric and laboratory measurements. Additionally, Kaplan‒Meier analysis was carried out among patients from the LIPDIOGRAM plus sub-study stratified by a decrease in body weight of 5% and 10% over 2 years of follow-up.

Kaplan‒Meier analysis was carried out to explore associations between changes in BMI and long-term prognosis. A 2-sided *p* < 0.05 was considered statistically significant.

### Ethics

The study protocol was approved by the Bioethical Committee of the Polish Chamber of Physicians (no. 51/2004/U) for 2004/2006 and by the Bioethical Commission of the District Medical Chamber in Częstochowa (no K.B.Cz.–0018/2015) for years 2015.

## Results

### Baseline characteristics

In the studied population of 45,615 patients, 10,987 (24.1%) were classified as normal weight, 19,134 (41.9%) were overweight and 320 (0.7%) patients had BMI below 18.5 kg/m2. Among the 15,174 (33.3%) patients with obesity, 11,117 (24.4%) were classified as having class I obesity, 3153 (6.9%) as having class II obesity, and 904 (2.0%) met the criteria for class III obesity. Baseline characteristics of the studied population are summarized in Table 1.

**Table 1:** Clinical characteristics of the study participants according to BMI categories

Variable	Underweight (n = 320^a^)	Normal weight (n = 10 987^a^)	Overweight (n = 19 134^a^)	Class 1 obesity (n = 11 117^a^)	Class 2 obesity (n = 3153^a^)	Class 3 obesity (n = 904^a^)	*p* value^b^
Age	49 (15)	53 (13)	57 (11)	58 (11)	58 (10)	57 (11)	< 0.001
Females	259 (81%)	7915 (72%)	10,908 (57%)	6396 (58%)	2045 (65%)	627 (69%)	< 0.001
BMI (kg/m^2^)	17.5 (0.9)	22.8 (1.6)	27.4 (1.4)	32.0 (1.4)	36.9 (1.4)	42.9 (2.6)	< 0.001
Higher education	202 (63%)	7429 (68%)	11,054 (58%)	5520 (50%)	1448 (46%)	378 (42%)	< 0.001
Urban place of residence	193 (60%)	6497 (59%)	10,793 (56%)	5810 (52%)	1552 (49%)	421 (47%)	< 0.001
WC (cm)	75 (12)	80 (9)	92 (9)	101 (9)	110 (11)	120 (14)	< 0.001
Metabolic syndrome	27 (8.4%)	1382 (13%)	6803 (36%)	6205 (56%)	2135 (68%)	664 (73%)	< 0.001
Smoking	106 (33%)	2875 (26%)	3432 (18%)	1685 (15%)	419 (13%)	105 (12%)	< 0.001
Diabetes Mellitus	13 (4.1%)	509 (4.6%)	1970 (10%)	2028 (18%)	848 (27%)	324 (36%)	< 0.001
Hypertension	95 (30%)	3436 (31%)	9630 (50%)	7232 (65%)	2372 (75%)	744 (82%)	< 0.001
Myocardial infarction	11 (3.4%)	443 (4.0%)	1236 (6.5%)	779 (7.0%)	192 (6.1%)	46 (5.1%)	< 0.001
Dyslipidemia	185 (58%)	7563 (69%)	14,920 (78%)	8763 (79%)	2425 (77%)	676 (75%)	< 0.001
Fibrate	8 (2.5%)	235 (2.1%)	672 (3.5%)	491 (4.4%)	147 (4.7%)	42 (4.6%)	< 0.001
Statin	52 (16%)	2251 (20%)	5612 (29%)	3713 (33%)	1090 (35%)	319 (35%)	< 0.001
TC (mmol/l)	5.44 (1.18)	5.57 (1.14)	5.62 (1.15)	5.53 (1.18)	5.41 (1.11)	5.29 (1.12)	< 0.001
LDL-C (mmol/l)	3.13 (1.00)	3.30 (0.99)	3.40 (1.00)	3.34 (1.00)	3.26 (0.95)	3.18 (0.96)	< 0.001
Non-HDL-C (mmol/l)	3.65 (1.13)	3.85 (1.08)	4.09 (1.09)	4.10 (1.11)	4.03 (1.04)	3.94 (1.04)	< 0.001
HDL-C (mmol/l)	1.79 (0.45)	1.72 (0.42)	1.53 (0.39)	1.43 (0.35)	1.39 (0.34)	1.35 (0.33)	< 0.001
TG (mmol/l)	1.26 (0.63)	1.34 (0.85)	1.67 (0.86)	1.89 (1.14)	1.95 (1.03)	1.97 (1.02)	< 0.001

*BMI* body mass index, *WC* waist circumference, *TC* total cholesterol, *LDL-C* low-density lipoprotein cholesterol, *HDL-C* high-density lipoprotein cholesterol, *TG* triglycerides, *Non-HDL-C* non-high-density lipoprotein cholesterol

^a^Mean (SD); n (%)

^b^Kruskal‒Wallis rank sum test; Pearson's Chi-squared test

There was a significant increase in the incidence of HTN, DM and dyslipidemia in both sexes as BMI increased (Table 1). In both female and male, a greater percentage of patients meeting the criteria for MetS across BMI categories was observed (see Additional file 1: Tables 1 and 2). In the entire cohort, as well as in female and male, the prevalence of smoking was highest in patients with the lowest BMI (Table 1; Additional file 1: Tables and 2). Patients with higher BMI more often lived in rural areas and had lower education levels compared with normal weight and underweight patients (Table 1; Additional file 1: Tables 1 and 2). In the entire cohort as well as among males, a history of MI was most prevalent in overweight and class I obese patients (Table 1; Additional file 1: Table 2). These groups also had the highest levels of LDL-C and TC in patients living with overweight and class I obesity in both the whole cohort and the male subpopulation (Table 1; Additional file 1: Table 2). Among females, the prevalence of previous MI was similar across overweight to class III obesity categories and was on average one-third higher than in underweight and normal weight patients (see Additional file 1: Table 1). Despite this, LDL-C levels differed by less than 0.4 mmol/L across BMI categories in the entire cohort in female as well as in male. HDL-C values decreased with increasing BMI, while TG levels increased with increasing BMI by 0.7 mmol/L between normal weight patients and those living with class III obesity. The lowest values of HDL-C and highest values of TG were observed in patients living with class III obesity (Table 1; Additional file 1: Tables 1 and 2). In general, the incidence of cardiovascular risk factors seen in both male and female individually follows trends found in the general population. They are presented in detail in Additional file 1: Tables 1 and 2.

### Survival analysis

Follow-up data were available for 44,620 patients. Within the median follow-up of 15.3 years, we observed 7559 (16.9%) deaths, including 60 (19.3%) for underweight, 1532 (14.3%) for normal weight 3085 (16.5%) for patients living with overweight and 1996 (18.3%), 651 (21.0%) and 235 (26.3%) for patients living with class I, II and III obesity, respectively.

**Fig. 1:**
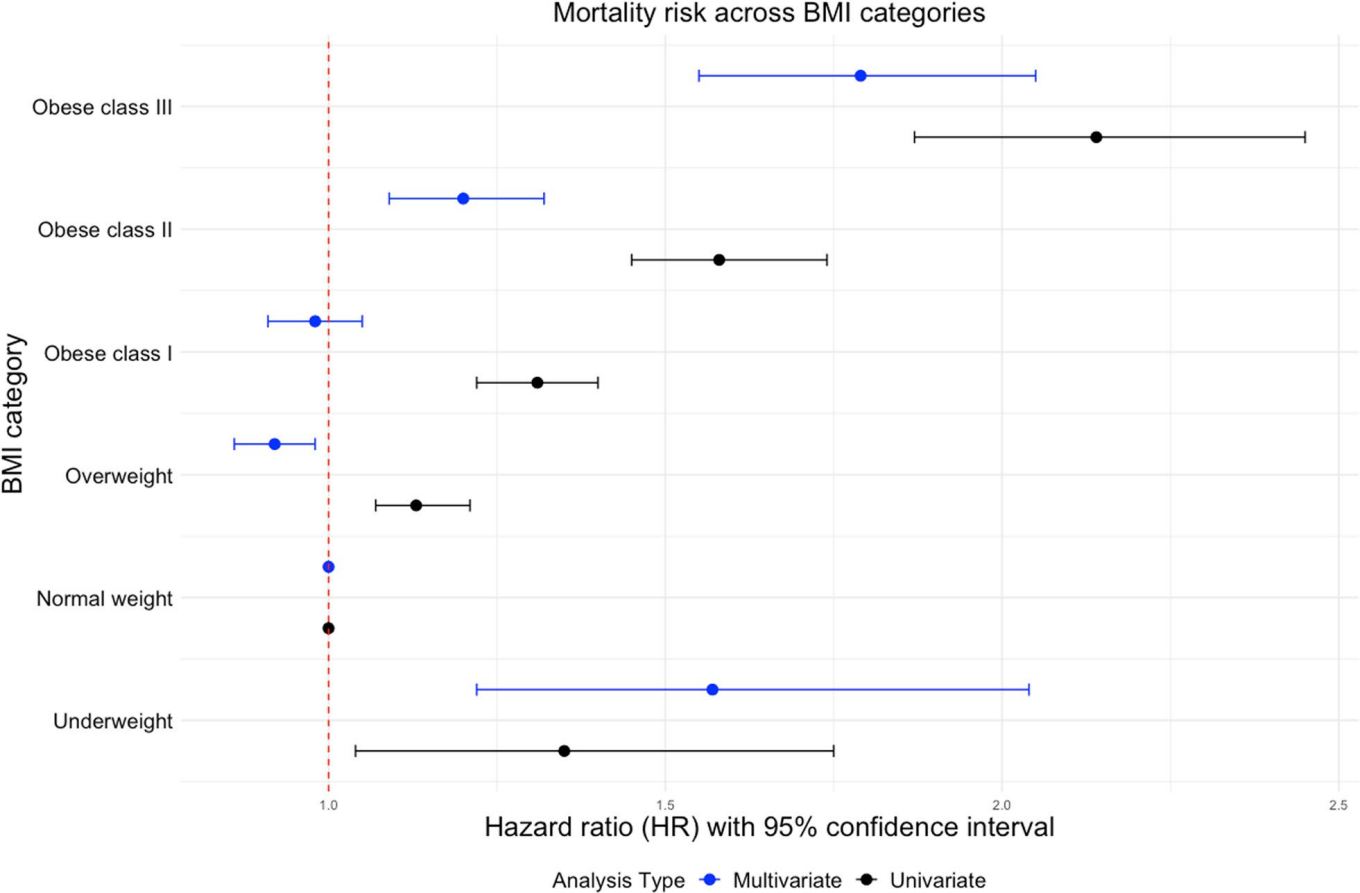
Forest plot of mortality risk across BMI categories. *BMI* body mass index, *HR* hazard ratio

**Fig. 2:**
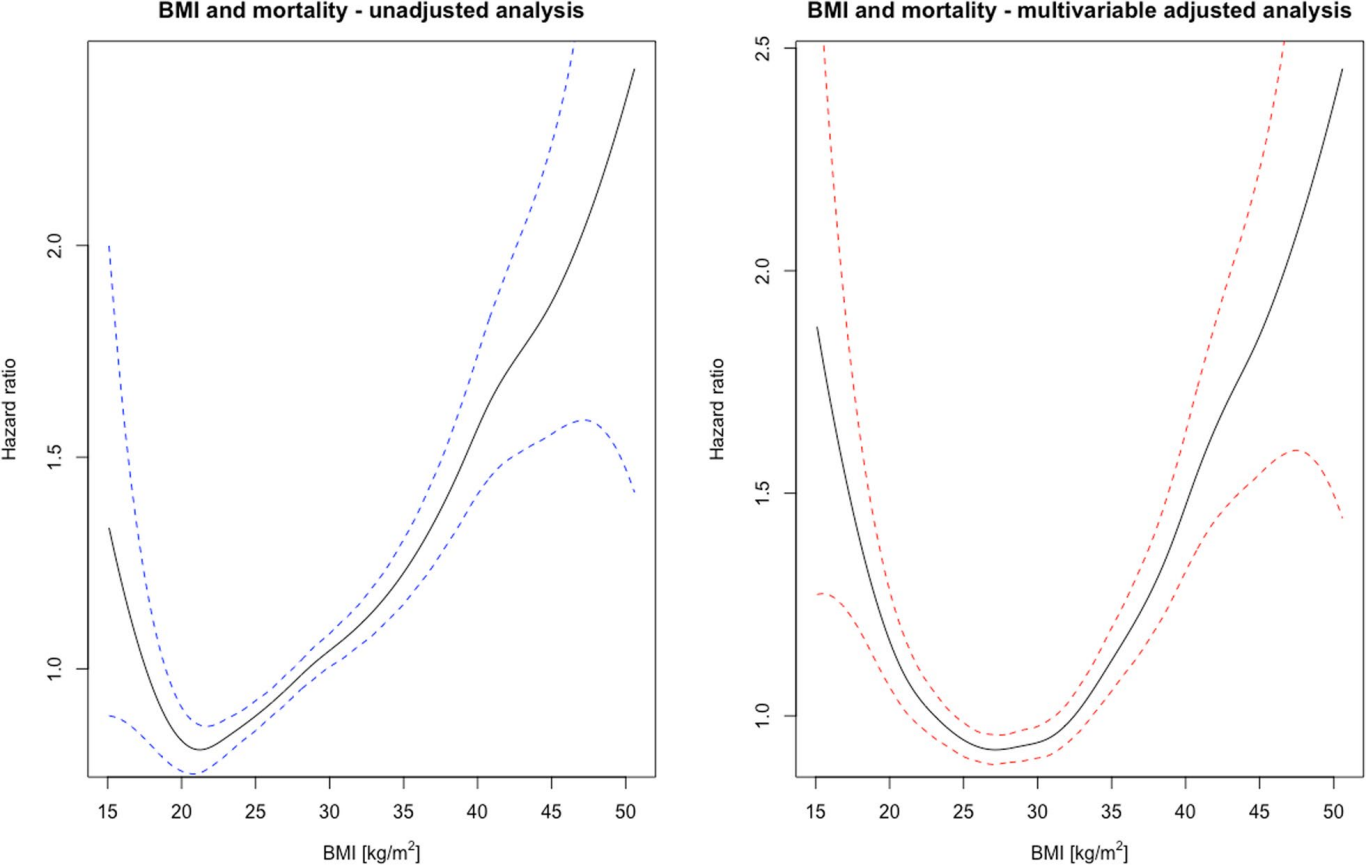
Association between BMI and mortality. **A** Unadjusted analysis and **B** adjusted analysis. *BMI* body mass index

In the univariate analysis, the lowest long-term mortality was observed in patients of normal weight. The risk of death during the entire observation period increased from a hazard ratio (HR) of 1.13 (95% confidence interval (CI) 1.07–1.21) for overweight patients through HR of 1.31 (95% CI, 1.22–1.40, *p* < 0.001) and 1.58 (95% CI, 1.45–1.74, *p* < 0.001) for class I and class II obesity respectively to an HR of 2.14 (95% CI, 1.87–2.45, *p* < 0.001) for patients living with class III obesity. In unadjusted analysis, patients who were underweight experienced similar mortality rates as patients with class I obesity (HR 1.35 (95% CI, 1.04–1.75, *p* < 0.001) (Fig. 1 and Fig. 2). Similar trend was observed in the female subgroup, but in the male group the lowest mortality was observed in overweight patients. (see Additional file 1: Figure 1). In a subgroup analysis of patients after MI and in patients suffering from DM lowest mortality was observed for overweight and obese patients. In subgroup of cigarette smokers and individuals with dyslipidemia relationship between BMI and mortality resembled this from general population (see Additional file 1: Figure 2). Interestingly in nonsmoking patients without comorbidities, the lowest mortality was observed for underweight and normal weight patients (see Additional file 1: Figure 3).

**Fig. 3:**
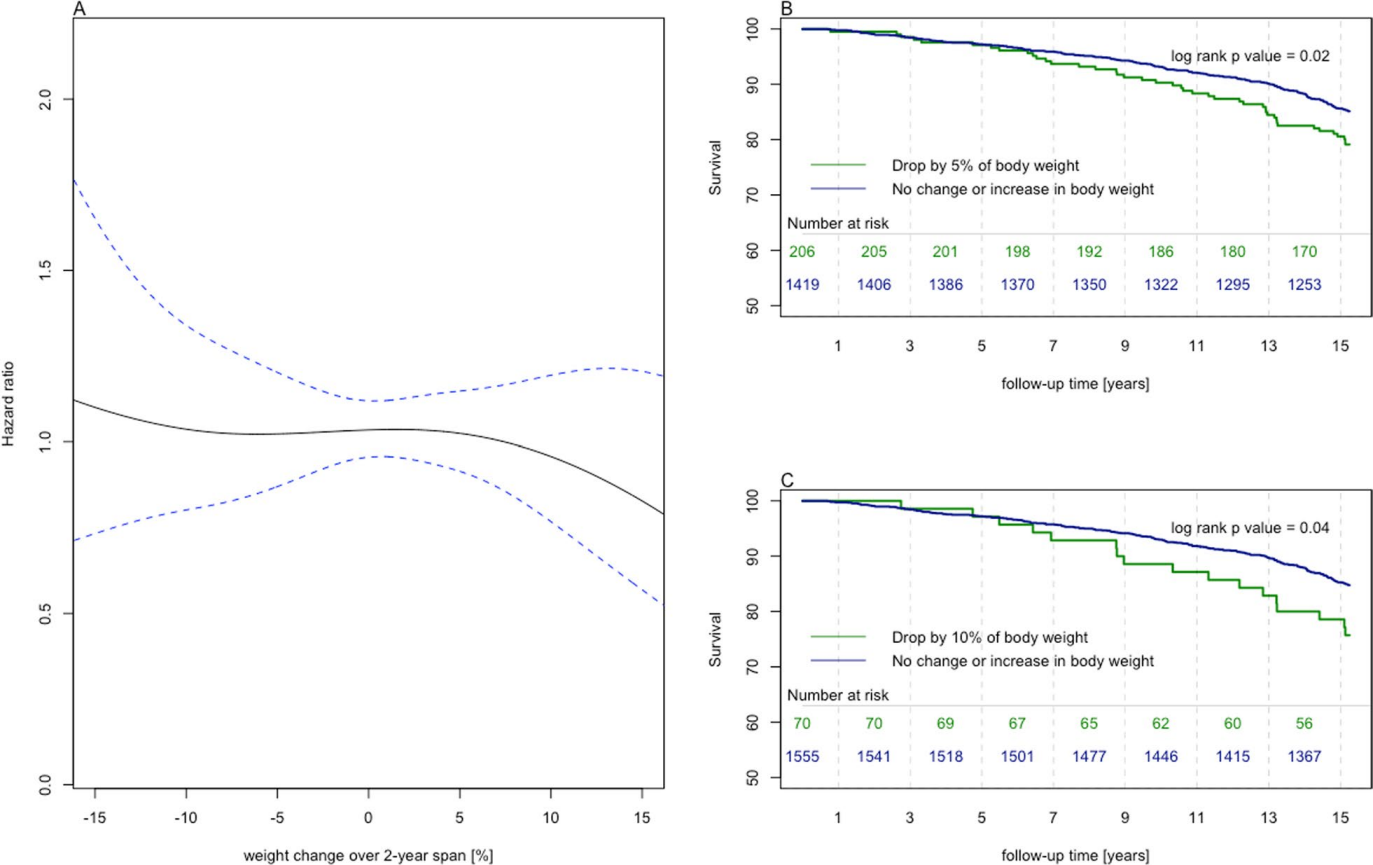
Effects of body weight change on mortality. **A** Spline curves of excess mortality according to body weight change. **B** Survival stratified by a 5% reduction in body weight. **C** Survival stratified by a 10% reduction in body weight

In the multivariate analysis, after adjustment for covariates, an apparent survival advantage was observed for overweight and class I obese patients—a “shift” in the BMI value for which mortality was the lowest could be observed, a similar result has been reported previously [20]. The highest mortality was observed for patients with class III obesity and those who were underweight (HR: 1.79, (95% CI 1.55–2.05, *p* < 0.001) and HR 1.57, (95% CI 1.22–2.04, *p* < 0.001), respectively) suggesting a U-shaped curve relationship (Figs. 1, 2). In the LIPIDOGRAM PLUS substudy, follow-up data on all-cause mortality were available for 1625 out of 1627 participants (99.9%). The analysis of this cohort showed a significant increase in the long-term mortality risk associated with a 5% and 10% reduction in body weight over two years. The mean weight loss in patients who experienced 5% reduction in body weight was 8.0 kg (SD-5.0 kg, min 3.0 kg max 37.0 kg) while the corresponding values in patients who lost 10% of their body weight were 12.4 kg (SD- 61 kg, min 5.0 kg max 37.0 kg). The corresponding HRs were 1.45 (95% CI 1.05–2.02, *p* = 0.03) for patients with at least 5% body weight reduction and 1.67 (95% CI 1.02–2.74, *p* < 0.001) for patients with at least 10% body weight reduction, respectively (Fig. 3). Notably, patients who experienced weight loss tended to have higher initial BMIs, be older, smoke more, have diabetes, and suffer from HTN more frequently (see Additional file 1: Tables 3 and 4).

## Discussion

In this analysis from a population of > 45 000 consecutive patients under the care of family physicians, BMI within the normal range was present in only one in four patients. Furthermore, one in three participants had a BMI > 30 kg/m^2^. When not adjusted for comorbidities, normal weight was associated with the lowest mortality. These results are consistent with a large meta-analysis on BMI and all-cause mortality including > 10 million participants, which also reported the lowest all-cause mortality for normal weight patients [21]. On the other hand, studies involving patients with chronic coronary artery disease [20, 22, 23], acute coronary syndromes [24], DM [25], heart failure [26], and cancer [27] reported the lowest mortality for overweight patients or obese patients and an increase in mortality for underweight patients and for subjects in class II or III obesity. In our subgroup analyses of patients after MI and patients with DM we also observed presence of obesity paradox with lowest mortality for overweight patients and patients living with class I obesity.

It was shown that a normal weight trajectory is associated with the greatest body weight gain in males younger than 40 years and females under 50 years of age, and peak weight is typically reached between 50 and 69 years of age [28, 29]. In most instances, subjects who experience weight loss do so unintentionally [28]. Therefore greater BMI may in fact be an indicator of better health status in those age groups as compared to BMI within normal range or below 18.5 kg/m^2^. It might be especially true in those observational and cross-sectional studies where we do not have data on weight loss trajectory before enrollment and lower BMI might often a result of an underlying ongoing disease rather than a change in lifestyle. In patients with end-stage chronic diseases, such as HF and cancer, a predominance of catabolic processes, loss of appetite, impairment of digestion resulting in caloric restriction and malnutrition contribute to increased mortality [29–36]. In patients with diabetes, weight loss is a marker of disease severity and loss of physical reserve and insufficient insulin secretion [25]. Therefore, the obesity paradox in advanced stages of cardiovascular diseases, cancers and diabetes should be explained by lower body weight and muscle wasting and even bone mass loss, which result in worse prognosis, not vice versa [37]. Others also observed that a change in body weight from overweight/obese to normal weight or underweight may result primarily from unintentional reduction of muscle mass [38, 39]. It raises doubts about any practical implications of the obesity paradox, except for the obvious necessity of searching for the underlying cause of weight loss. This is also in line with the results of our study, as the obesity paradox was present in subgroups of patients with MI and DM while in general cohort it was observed only after adjustment for comorbidities. More importantly patients who lost weight were more burdened with comorbidities and in non-smoking patients without comorbidities mortality benefit was observed even in lower end of normal weight category.

The results of other studies also show that intentional weight loss (including bariatric surgery) might be associated with mortality benefit and reduction of cardiovascular events but unintentional weight loss is not [40, 41]. Most of observational studies, including ours, do not analyze if the weight loss was intentional or not which may greatly affect conclusions regarding obesity paradox. Intentional weight loss, and incorporating lifestyle changes is challenging and for many difficult to maintain. Studies from the same period as the LIPIDOGRAM PLUS study, show that, approximately 1/3 of patients analyzed in cross sectional studies undertake some action to lose body weight [28, 42]. Of these, only 20% are successful at sustaining a lower weight. The remaining two thirds do not engage in any action to lower body weight and despite this, as much as 30% experience unintentional weight loss [28, 42]. Recent data from the STEP1 (Effect and Safety of Semaglutide 2.4 mg once weekly in Subjects With Overweight or Obesity) trial show that one year after cessation of treatment with semaglutide, participants regained 2/3 of their prior weight loss [43].

On the basis of data from other epidemiological studies as well as from the fact that the obesity paradox appeared after adjustment for clinical characteristic including comorbidities and that weight loss was associated with worse long-term prognosis, we might assume that in most patients, weight loss was unintentional. As mentioned earlier the concept of the obesity paradox was seen mostly in observational studies that in vast majority do not have information on whether body weight loss was intentional or not [24, 44, 45].

Another possible cause of the high mortality among underweight people in our study is the high percentage of smokers in this group. Numerous studies indicate that the primary factor in the increase in mortality among smokers is lung cancer [46, 47]. In addition, because nicotine hinders appetite, it is easier for smokers to maintain their BMI and not gain weight [48, 49]. The fear of gaining weight is also one of the factors that make it difficult to quit smoking [50]. The results of our analysis showed that in nonsmokers without DM or a history of MI, the risk of mortality was lowest among patients with BMI within lower end of normal body weight category, and it increased with increasing BMI. Similarly, Veronese et al. showed that people with a BMI within the normal range (18.5–22.4 kg/m^2^), maintaining a healthy diet, engaging in physical activity, moderation in the use of stimulants, and not smoking, had the lowest risk of premature death [51].

**Fig. 4:**
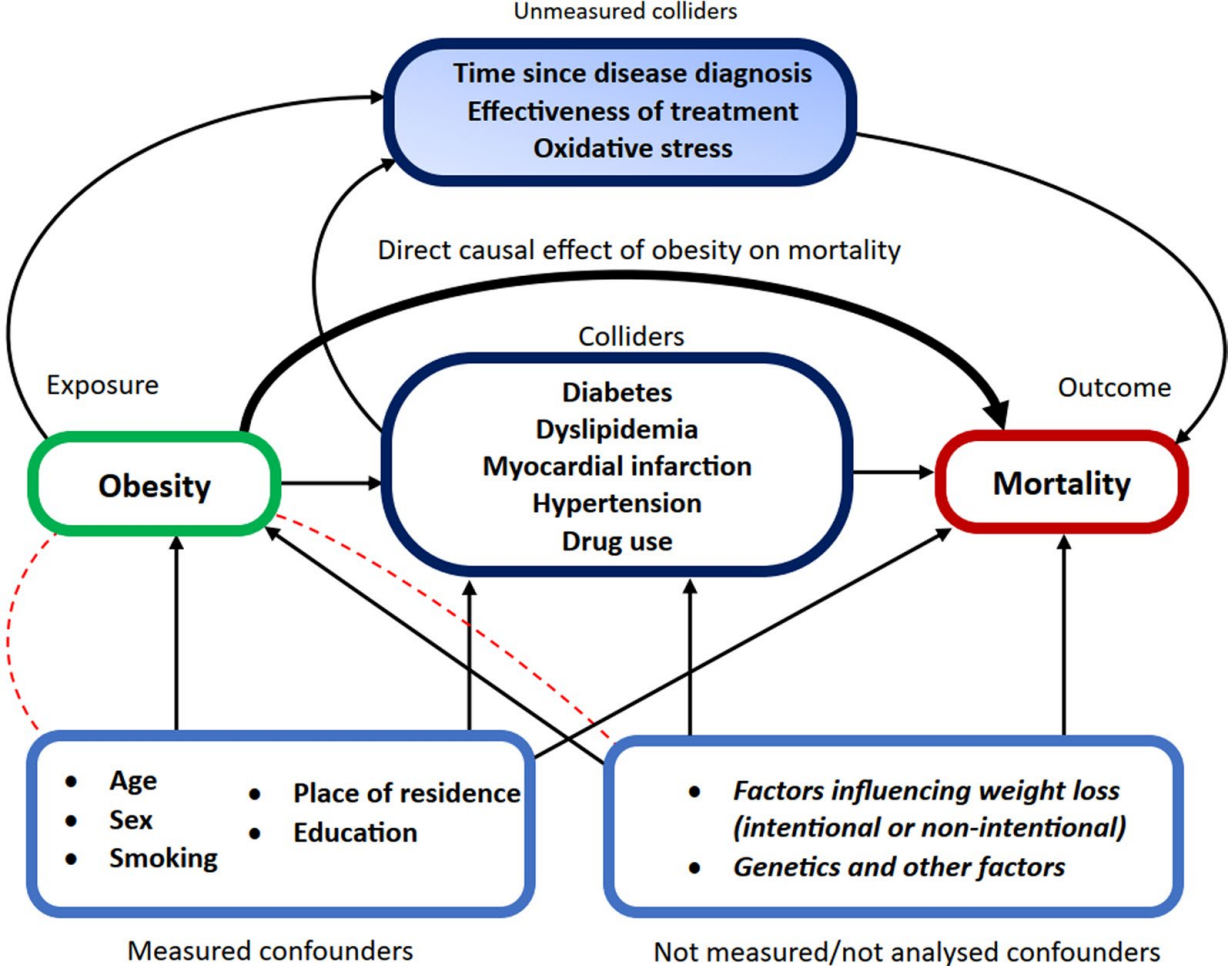
Collider stratification bias as an explanation of obesity paradox. A directed acyclic graph representing the relations between obesity (BMI), cardiometabolic mediators and mortality. Dashed red lines represent correlations created by conditioning/adjusting on colliders. The true causal effect of obesity on mortality may be largely mediated by cardiometabolic diseases. As those diseases can also be influenced by factors other than BMI, they are colliders. Subgroup analyses involving patients with or without cardiometabolic diseases are subject to collider bias as non-causal correlations between confounders and obesity are created

In our study, obesity paradox is indeed apparent in adjusted analysis and subgroup analyses (Additional file 1: Figures 2 and 3). Nevertheless, some superficial “beneficial” effects of obesity in patients with comorbidities can be at least partially explained by collider stratification bias [52, 53]. Colliders are factors that are correlated with both the exposure (BMI) and other factors. Collider stratification bias occurs when study inclusion criteria, adjustment or stratification depend on the collider(s). This creates a non-causal correlation between the exposure and factors that affect the collider [52, 54]. It may partially explain that obesity paradox was observed in studies including only patients with certain diseases i.e. diabetes, heart failure etc. but less often in general populations. In case of our work, colliders would be those conditions whose frequency increases with increasing BMI such as previous MI, DM, dyslipidemia, HTN. At the same time, occurrence of those colliders is also influenced by factors such as smoking, sex, education, place of residence or non-measured factors such as genetic influences. An example of collider bias may be a situation in which MI (collider) is caused by obesity and/or smoking. In this case, in the subgroup of patients defined by the collider (previous MI) those with a higher BMI would have an observed tendency not to have other factors that predispose towards MI. In this case higher BMI would appear protective, as in the subgroup defined by collider, it would be negatively correlated with factors like smoking, which may have a more negative impact on survival than BMI itself [12]. We are also the opinion that unmeasured variables such as time elapsed from the occurrence of obesity to the enrollment of participants and intentionality of body weight reduction are the two most important unmeasured confounders missing in our and other observational studies that evaluate the relationship between obesity and mortality (Fig. 4).

## Study strengths and limitations

The main strength of this study is the inclusion of a large number of patients. Importantly, patients involved in the study were recruited from all 16 regions of Poland and were representative of the primary health care population. Another strength of the study is that all the biochemical measurements were conducted in a central laboratory, which conforms to all the required quality control standards; this ensures the reliability of the results. Medical history and office measurements were collected by doctors who knew the patients and looked after them on a regular basis. In 2004 and 2006, the data gathered were the same, but in 2015 this was extended to include blood pressure, heart rate and blood glucose.

A limitation of the study is that it was conducted in only one country. Primary healthcare practices were selected at random, but physicians enrolled patients consecutively. Moreover, we did not gain access to data on the causes of death of patients; therefore, we conducted our analysis based on all-cause death. Data pertaining to factors that influenced weight change in each patient were also unavailable. Additionally, although BMI is a popular measure of obesity, other indices also exist, some of which could be less prone to “obesity paradox”. While it remains possible that some beneficial effects of obesity on survival exist in some populations, this would need to be contrary to the trend in the overall population. Nevertheless, if those populations suffer from conditions caused in part by obesity, observational studies like this one are difficult to interpret due to potential for collider bias and factors that influence both weight and mortality.

In medical sciences, counterintuitive results—such as those from the ACCORD Study on aggressive glycemic control in diabetes or the Minnesota Coronary Experiment—can lead to important discoveries [55, 56]. We therefore acknowledge the possibility of some protective effects of obesity, as recently postulated by the increased levels of plasminogen activator inhibitor 1 in obese patients with MI, which we were not able to test in this analysis [57].

## Conclusions

Being underweight, overweight or obese is associated with higher mortality risk in a population of patients in primary care. Patients who lost weight were older and more burdened with cardiometabolic diseases which may suggest unintentional weight loss and were at higher risk of death in the long-term follow-up. In nonsmoking patients without comorbidities, the lowest mortality was observed in those with a BMI < 25 kg/m^2^ and mortality benefit was observed even in the lower range of normal weight interval and no U-curve relationship was observed. Obesity paradox should not be a reason to advocate possible benefits of weight gain in normal weight individuals.

### Supplementary Information

**Additional file 1.** Clinical characteristics of patients by sex; clinical characteristics of patients by weight loss experienced, mortality hazard ratio versus BMI in sex and comorbidity subgroups as well as patients without comorbidities..

## Data Availability

The datasets generated during and/or analyzed during the current study are available from the corresponding author on reasonable request.
