# Supplementary material for: Association of body mass index and long-term mortality in patients from nationwide LIPIDOGRAM 2004–2015 cohort studies: no obesity paradox?
Source: Cardiovasc Diabetol. 2023 Nov 28;22:323. doi: 10.1186/s12933-023-02059-0 (PMC10685602; doi:10.1186/s12933-023-02059-0)
Supplement: Supplementary file 1 — Additional file 1. Clinical characteristics of patients by sex; clinical characteristics of patients by weight loss experienced, mortality hazard ratio versus BMI in sex and comorbidity subgroups as well as patients without comorbidities.. [file 12933_2023_2059_MOESM1_ESM.docx]

**SUPPLEMENTARY MATERIAL**

**Supplementary Table 1.** Clinical characteristics of females according to BMI categories.

| **Variable** | **Underweight**   n = 259^1^ | **Normal weight**   n = 7915^1^ | **Overweight**   n = 10 908^1^ | **Class 1 obesity**   n = 6396^1^ | **Class 2 obesity**   n = 2045^1^ | **Class 3 obesity**   n = 627^1^ | **p-value**^2^ |
| --- | --- | --- | --- | --- | --- | --- | --- |
| Age, years, Mean (SD) | 47 (15) | 53 (13) | 58 (11) | 59 (11) | 59 (10) | 57 (11) | <0.001 |
| BMI [kg/m^2^] | 17.6 (0.8) | 22.7 (1.6) | 27.4 (1.4) | 32.0 (1.4) | 36.9 (1.4) | 42.9 (2.6) | <0.001 |
| WC [cm] | 73 (12) | 78 (8) | 89 (9) | 99 (9) | 108 (10) | 117 (12) | <0.001 |
| Higher education | 177 (68%) | 5723 (72%) | 6485 (59%) | 3149 (49%) | 936 (46%) | 267 (43%) | <0.001 |
| Urban place of residence | 157 (61%) | 4769 (60%) | 6103 (56%) | 3357 (52%) | 1002 (49%) | 290 (46%) | <0.001 |
| Metabolic syndrome | 26 (10%) | 1076 (14%) | 4176 (38%) | 3550 (56%) | 1355 (66%) | 462 (74%) | <0.001 |
| Smoking | 79 (31%) | 1814 (23%) | 1661 (15%) | 753 (12%) | 221 (11%) | 67 (11%) | <0.001 |
| Diabetes Mellitus | 10 (3.9%) | 315 (4.0%) | 1031 (9.5%) | 1125 (18%) | 521 (25%) | 214 (34%) | <0.001 |
| Hypertension | 79 (31%) | 2405 (30%) | 5586 (51%) | 4235 (66%) | 1532 (75%) | 516 (82%) | <0.001 |
| Myocardial infarction | 6 (2.3%) | 174 (2.2%) | 356 (3.3%) | 245 (3.8%) | 79 (3.9%) | 23 (3.7%) | <0.001 |
| Dyslipidemia | 149 (58%) | 5397 (68%) | 8521 (78%) | 5109 (80%) | 1579 (77%) | 480 (77%) | <0.001 |
| Fibrate | 7 (2.7%) | 150 (1.9%) | 327 (3.0%) | 243 (3.8%) | 88 (4.3%) | 25 (4.0%) | <0.001 |
| Statin | 38 (15%) | 1575 (20%) | 3198 (29%) | 2144 (34%) | 692 (34%) | 218 (35%) | <0.001 |
| TC [mmol/l] | 5.51 (1.17) | 5.61 (1.13) | 5.71 (1.15) | 5.63 (1.17) | 5.48 (1.11) | 5.35 (1.12) | <0.001 |
| LDL-C [mmol/l] | 3.16 (1.01) | 3.30 (0.99) | 3.43 (1.00) | 3.40 (1.02) | 3.29 (0.97) | 3.23 (0.97) | <0.001 |
| non-HDL-C [mmol/l] | 3.68 (1.13) | 3.83 (1.08) | 4.09 (1.09) | 4.11 (1.12) | 4.02 (1.04) | 3.95 (1.06) | <0.001 |
| HDL-C [mmol/l] | 1.83 (0.45) | 1.78 (0.41) | 1.63 (0.39) | 1.52 (0.35) | 1.46 (0.33) | 1.40 (0.33) | <0.001 |
| TG [mmol/l] | 1.24 (0.62) | 1.30 (0.82) | 1.59 (0.77) | 1.77 (0.82) | 1.84 (0.88) | 1.89 (0.83) | <0.001 |

^1^Mean (SD); n (%),^2^Kruskal-Wallis rank sum test; Pearson's Chi-squared test BMI - Body Mass Index, WC - Waist Circumference, TC - Total Cholesterol, LDL-C - Low-Density Lipoprotein Cholesterol, HDL-C - High-Density Lipoprotein Cholesterol, TG - Triglycerides, Non-HDL-C - Non-High-Density Lipoprotein Cholesterol.

**Supplementary Table 2.** Clinical characteristics of males according to BMI categories

| **Variable** | **Underweight**   n = 61^1^ | **Normal weight**   n = 3072^1^ | **Overweight**   n = 8226^1^ | **Class 1 obesity**   n = 4721^1^ | **Class 2 obesity**   n = 1108^1^ | **Class 3 obesity**   n = 277^1^ | **p-value**^2^ |
| --- | --- | --- | --- | --- | --- | --- | --- |
| Age | 57 (14) | 55 (13) | 56 (12) | 56 (11) | 56 (10) | 55 (10) | <0.001 |
| BMI [kg/m^2^] | 17.1 (1.0) | 23.2 (1.4) | 27.5 (1.4) | 32.0 (1.4) | 36.8 (1.3) | 42.7 (2.5) | <0.001 |
| WC [cm] | 82 (11) | 87 (8) | 96 (8) | 105 (8) | 115 (10) | 126 (15) | <0.001 |
| Higher education | 25 (41%) | 1706 (56%) | 4569 (56%) | 2371 (50%) | 512 (46%) | 111 (40%) | <0.001 |
| Urban place of residence | 36 (59%) | 1728 (56%) | 4690 (57%) | 2453 (52%) | 550 (50%) | 131 (47%) | <0.001 |
| Metabolic syndrome | 1 (1.6%) | 306 (10.0%) | 2627 (32%) | 2655 (56%) | 780 (70%) | 202 (73%) | <0.001 |
| Smoking | 27 (44%) | 1061 (35%) | 1771 (22%) | 932 (20%) | 198 (18%) | 38 (14%) | <0.001 |
| Diabetes Mellitus | 3 (4.9%) | 194 (6.3%) | 939 (11%) | 903 (19%) | 327 (30%) | 110 (40%) | <0.001 |
| Hypertension | 16 (26%) | 1031 (34%) | 4044 (49%) | 2997 (63%) | 840 (76%) | 228 (82%) | <0.001 |
| Myocardial infarction | 5 (8.2%) | 269 (8.8%) | 880 (11%) | 534 (11%) | 113 (10%) | 23 (8.3%) | 0.009 |
| Dyslipidemia | 36 (59%) | 2166 (71%) | 6399 (78%) | 3654 (77%) | 846 (76%) | 196 (71%) | <0.001 |
| Fibrate | 1 (1.6%) | 85 (2.8%) | 345 (4.2%) | 248 (5.3%) | 59 (5.3%) | 17 (6.1%) | <0.001 |
| Statin | 14 (23%) | 676 (22%) | 2414 (29%) | 1569 (33%) | 398 (36%) | 101 (36%) | <0.001 |
| TC [mmol/l] | 5.17 (1.19) | 5.48 (1.16) | 5.50 (1.14) | 5.39 (1.16) | 5.28 (1.10) | 5.16 (1.09) | <0.001 |
| LDL-C [mmol/l] | 2.98 (0.94) | 3.32 (1.00) | 3.36 (0.98) | 3.25 (0.97) | 3.21 (0.93) | 3.08 (0.95) | <0.001 |
| non-HDL [mmol/l] | 3.56 (1.11) | 3.92 (1.09) | 4.09 (1.08) | 4.09 (1.11) | 4.04 (1.03) | 3.93 (1.01) | <0.001 |
| HDL-C [mmol/l] | 1.61 (0.44) | 1.56 (0.41) | 1.41 (0.35) | 1.30 (0.32) | 1.25 (0.31) | 1.23 (0.30) | <0.001 |
| Trig [mmol/l] | 1.35 (0.68) | 1.46 (0.92) | 1.76 (0.96) | 2.05 (1.45) | 2.14 (1.24) | 2.15 (1.34) | <0.001 |

^1^Mean (SD); n (%),^2^Kruskal-Wallis rank sum test; Pearson's Chi-squared test BMI - Body Mass Index, WC - Waist Circumference, TC - Total Cholesterol, LDL-C - Low-Density Lipoprotein Cholesterol, HDL-C - High-Density Lipoprotein Cholesterol, TG - Triglycerides, Non-HDL-C - Non-High-Density Lipoprotein Cholesterol.

**Supplementary Table 3.** Clinical characteristics of patients who experienced a drop of body weight by 5 %. (LIPIDOGRAM plus study).

| **Characteristic** | **Drop by 5% of body weight**, n = 206 | **No change or increase in body weight**, n = 1,419 | **p-value**^1^ |
| --- | --- | --- | --- |
| Age, years, Mean (SD) | 55 (10) | 53 (9) | 0.003 |
| Females, n (%) | 132 (64%) | 882 (62%) | 0.59 |
| BMI [kg/m^2^], Mean (SD) | 29.8 (4.7) | 27.6 (4.5) | <0.001 |
| WC [cm], Mean (SD) | 93 (14) | 91 (13) | 0.023 |
| Higher education, n (%) | 115 (56%) | 967 (68%) | <0.001 |
| Urban place of residence, n (%) | 104 (50%) | 747 (53%) | 0.56 |
| Metabolic syndrome, n (%) | 62 (30%) | 324 (23%) | 0.022 |
| Smoking, n (%) | 49 (24%) | 263 (19%) | 0.074 |
| Diabetes Mellitus, n (%) | 31 (15%) | 117 (8.2%) | 0.002 |
| Hypertension, n (%) | 105 (51%) | 596 (42%) | 0.015 |
| Myocardial infarction, n (%) | 11 (5.3%) | 70 (4.9%) | 0.80 |
| Dyslipidemia, n (%) | 159 (77%) | 1,049 (74%) | 0.3 |
| Fibrate, n (%) | 4 (1.9%) | 61 (4.3%) | 0.11 |
| Statin, n (%) | 56 (27%) | 344 (24%) | 0.36 |
| Total Cholesterol [mmol/l], Mean (SD) | 5.86 (1.02) | 5.82 (1.16) | 0.32 |
| LDL-C [mmol/l], Mean (SD) | 3.44 (0.86) | 3.38 (1.00) | 0.22 |
| non-HDL-C [mmol/l], Mean (SD) | 4.19 (0.95) | 4.13 (1.10) | 0.20 |
| HDL-C [mmol/l], Mean (SD) | 1.67 (0.38) | 1.69 (0.40) | 0.73 |
| TG [mmol/l], Mean (SD) | 1.64 (0.77) | 1.63 (0.80) | 0.49 |

^1^Wilcoxon rank sum test; Pearson's Chi-squared test BMI - Body Mass Index, WC - Waist Circumference, TC - Total Cholesterol, LDL-C - Low-Density Lipoprotein Cholesterol, HDL-C - High-Density Lipoprotein Cholesterol, TG - Triglycerides, Non-HDL-C - Non-High-Density Lipoprotein Cholesterol.

**Supplementary Table 4.** Clinical characteristics of patients who experienced drop of body weight by 10%. (LIPIDOGRAM plus study).

| **Characteristic** | **Drop by 10% of body weight**, n = 70 | **No change or increase in body weight**, n = 1,555 | **p-value**^1^ |
| --- | --- | --- | --- |
| Age, years, Mean (SD) | 56 (11) | 53 (9) | 0.002 |
| Females, n (%) | 49 (70%) | 965 (62%) | 0.18 |
| BMI [kg/m^2^], Mean (SD) | 30.5 (4.5) | 27.8 (4.5) | <0.001 |
| WC [cm], Mean (SD) | 92 (13) | 91 (13) | 0.29 |
| Higher education, n (%) | 35 (50%) | 1,047 (67%) | 0.003 |
| Urban place of residence, n (%) | 34 (49%) | 817 (53%) | 0.52 |
| Metabolic syndrome, n (%) | 21 (30%) | 365 (23%) | 0.21 |
| Smoking, n (%) | 16 (23%) | 296 (19%) | 0.43 |
| Diabetes Mellitus, n (%) | 11 (16%) | 137 (8.8%) | 0.050 |
| Hypertension, n (%) | 42 (60%) | 659 (42%) | 0.004 |
| Myocardial infarction, n (%) | 5 (7.1%) | 76 (4.9%) | 0.39 |
| Dyslipidemia, n (%) | 52 (74%) | 1,156 (74%) | 0.99 |
| Fibrate, n (%) | 0 (0%) | 65 (4.2%) | 0.11 |
| Statin, n (%) | 21 (30%) | 379 (24%) | 0.29 |
| Total Cholesterol [mmol/l], Mean (SD) | 5.81 (1.06) | 5.82 (1.15) | 0.97 |
| LDL-C [mmol/l], Mean (SD) | 3.37 (0.90) | 3.39 (0.99) | 0.98 |
| HDL-C [mmol/l], Mean (SD) | 1.74 (0.39) | 1.69 (0.40) | 0.26 |
| non-HDL-C [mmol/l], Mean (SD) | 4.07 (1.00) | 4.14 (1.09) | 0.84 |
| TG [mmol/l], Mean (SD) | 1.53 (0.72) | 1.63 (0.80) | 0.43 |

^1^Wilcoxon rank sum test; Pearson's Chi-squared test; Fisher's exact test BMI - Body Mass Index, WC - Waist Circumference, TC - Total Cholesterol, LDL-C - Low-Density Lipoprotein Cholesterol, HDL-C - High-Density Lipoprotein Cholesterol, TG - Triglycerides, Non-HDL-C - Non-High-Density Lipoprotein Cholesterol.


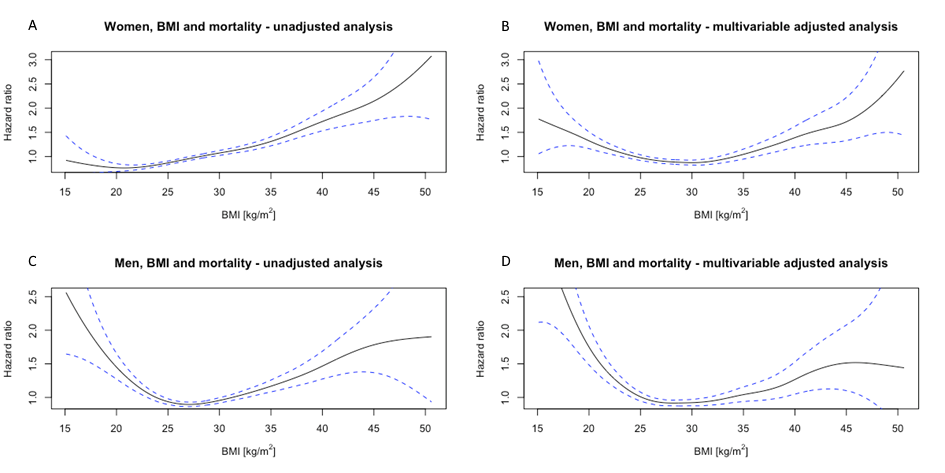


**Supplementary Figure 1.** Unadjusted curves for all-cause mortality according to BMI in females (A) and males (C). Multivariable-adjusted curves for all-cause mortality according to BMI in females (B) and males (D). BMI – body mass index.


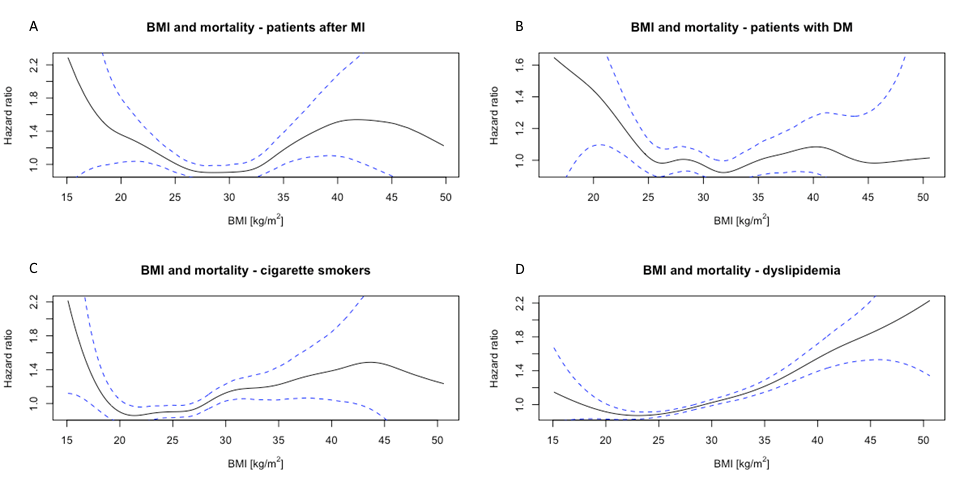


**Supplementary Figure 2.** Unadjusted curves for all-cause mortality according to the BMI in patients after MI (A), patients with DM (B), cigarette smokers (C), and patients with dyslipidemia (D). BMI= body mass index, MI= myocardial infarction, DM= diabetes mellitus.


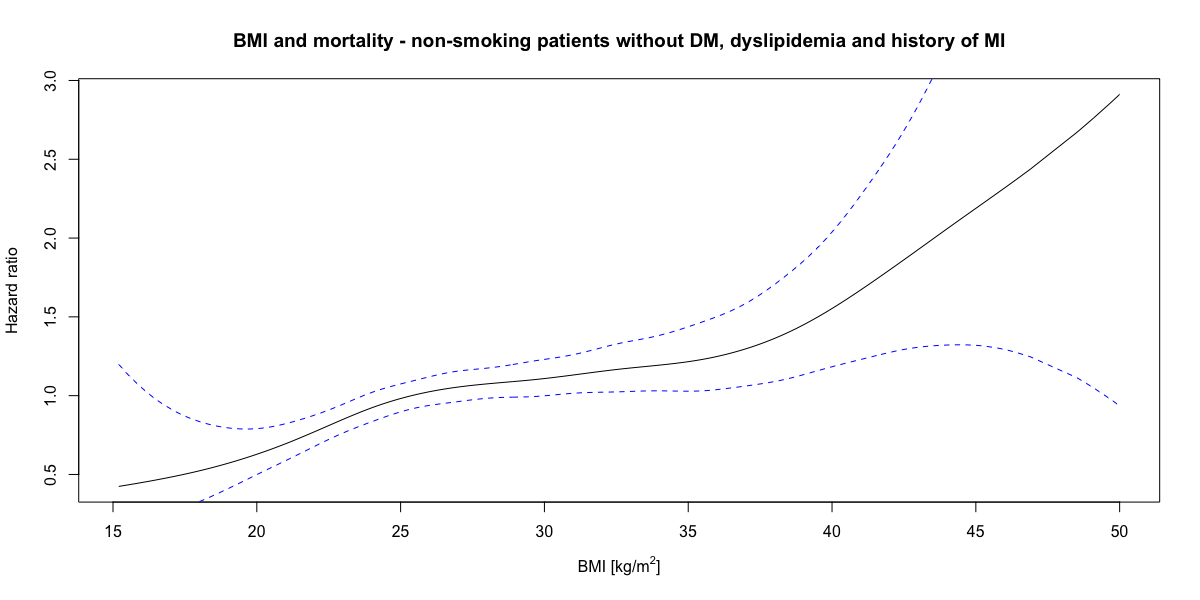


**Supplementary Figure 3.** Unadjusted curves for all-cause mortality according to the BMI in non-smoking patients without DM, dyslipidemia and MI). BMI= body mass index, DM= diabetes mellitus, MI= myocardial infarction
